# An Unusual Case of Multiple Left Ventricular Aneurysms Masquerading as Diverticula in the Setting of Myocardial Infarction

**DOI:** 10.1177/11795468211006698

**Published:** 2021-03-27

**Authors:** Hussain Alzayer, Ahmad Alshatti, Akeel Alali

**Affiliations:** 1 Division of Cardiology, Hamilton General Hospital, McMaster University, Hamilton Health Sciences, Hamilton, ON, Canada; 2 Department of Radiology, King Saud Bin Abdulaziz University for Health Sciences, Riyadh, Kingdom of Saudi Arabia

**Keywords:** Aneurysm, diverticula, myocardial infarction

## Abstract

The distinction between cardiac aneurysms and diverticula can be very difficult by angiography. Left ventricular (LV) aneurysms usually occur following transmural myocardial infarction. On the other hand, cardiac diverticula are most commonly congenital. They are commonly detected by cardiac CT with a prevalence of 2.2%. Here we present a case of a 60-year-old male with the incidental finding of multiple LV aneurysms masquerading as diverticula in the setting of myocardial infarction with near normal coronary arteries. Moreover, this case highlights the limitation of coronary angiography in the diagnosis of myocardial infarction with no obstructive atherosclerosis (MINOCA).

## Case Vignette

A 60-year-old male with cardiac risk factors in the form of smoking and premature family history of coronary artery disease and no previous cardiac history, who presented to our facility with acute onset of retrosternal chest pain lasting for 30 minutes with radiation to the left arm. He denied any other symptoms. His medications included suboxone and ibuprofen.

On examination his temperature was 36.9°C, blood pressure 161/94 and heart rate 86 beats per minute and regular. His cardiorespiratory examination was within normal limits with no evidence of congestive heart failure.

His investigations showed a white count of 10.6 × 10^9^/L, hemoglobin 168 g/L, platelet count 339 × 10^9^/L, his high sensitivity troponin peaked at 2997 ng/L (normal reference cutoff ⩽30 ng/L). He had normal kidney function and coagulation profile. His initial electrocardiogram showed sinus rhythm with left axis deviation and Q waves in leads V1 to V2 (Supplemental Figure 1).

**Figure 1.**
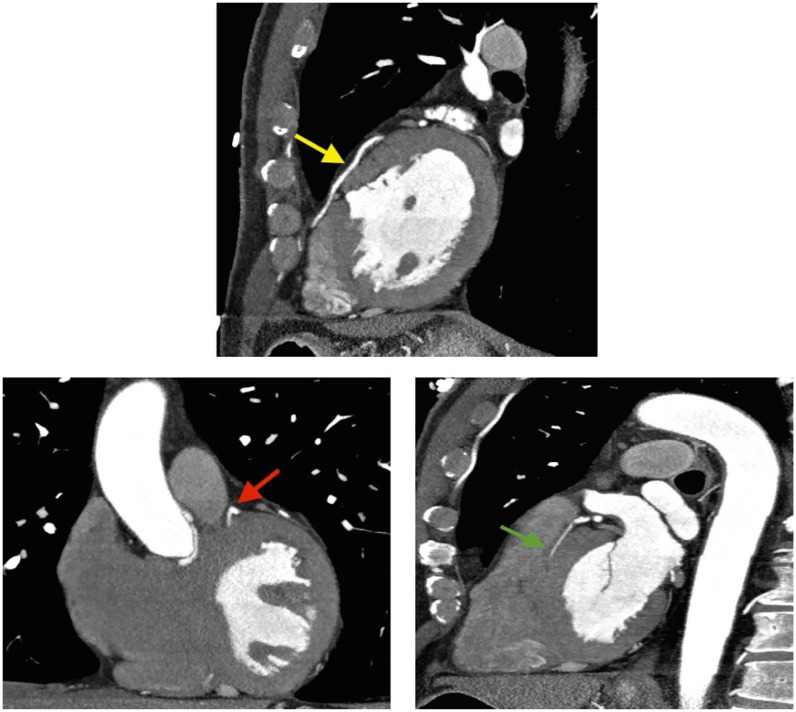
Coronary CTA with multiplanar reformatted images of the left anterior descending artery (LAD) and the diagonal branch. The LAD is a large vessel which is free of significant stenosis (Yellow arrow). There is 1 diagonal branch which has small caliber (<1 mm). The origin of this diagonal branch is patent with no plaques (red arrow). This vessel terminates abruptly raising the possibility of a small mid to distal occluded segment (green arrow).

**Figure 2.**
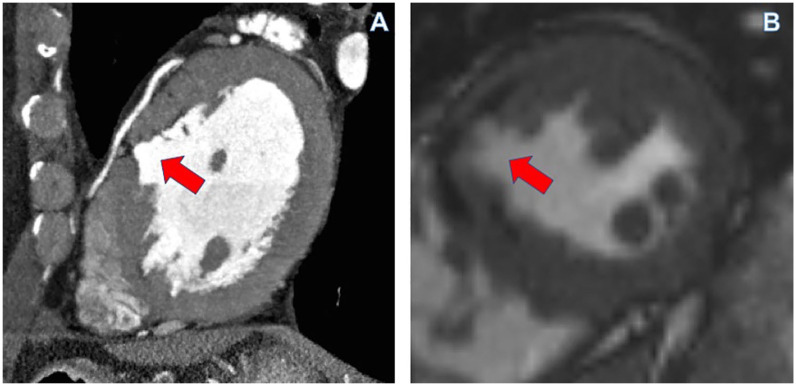
(A) Enhanced cardiac CT sagittal view shows focal aneurysm in the mid septal segment of the left ventricle (arrow) with associated minimal fatty infiltration and (B) cardiac MR short axis view confirms the presence of focal aneurysm as demonstrated on the cardiac CT.

**Figure 3.**
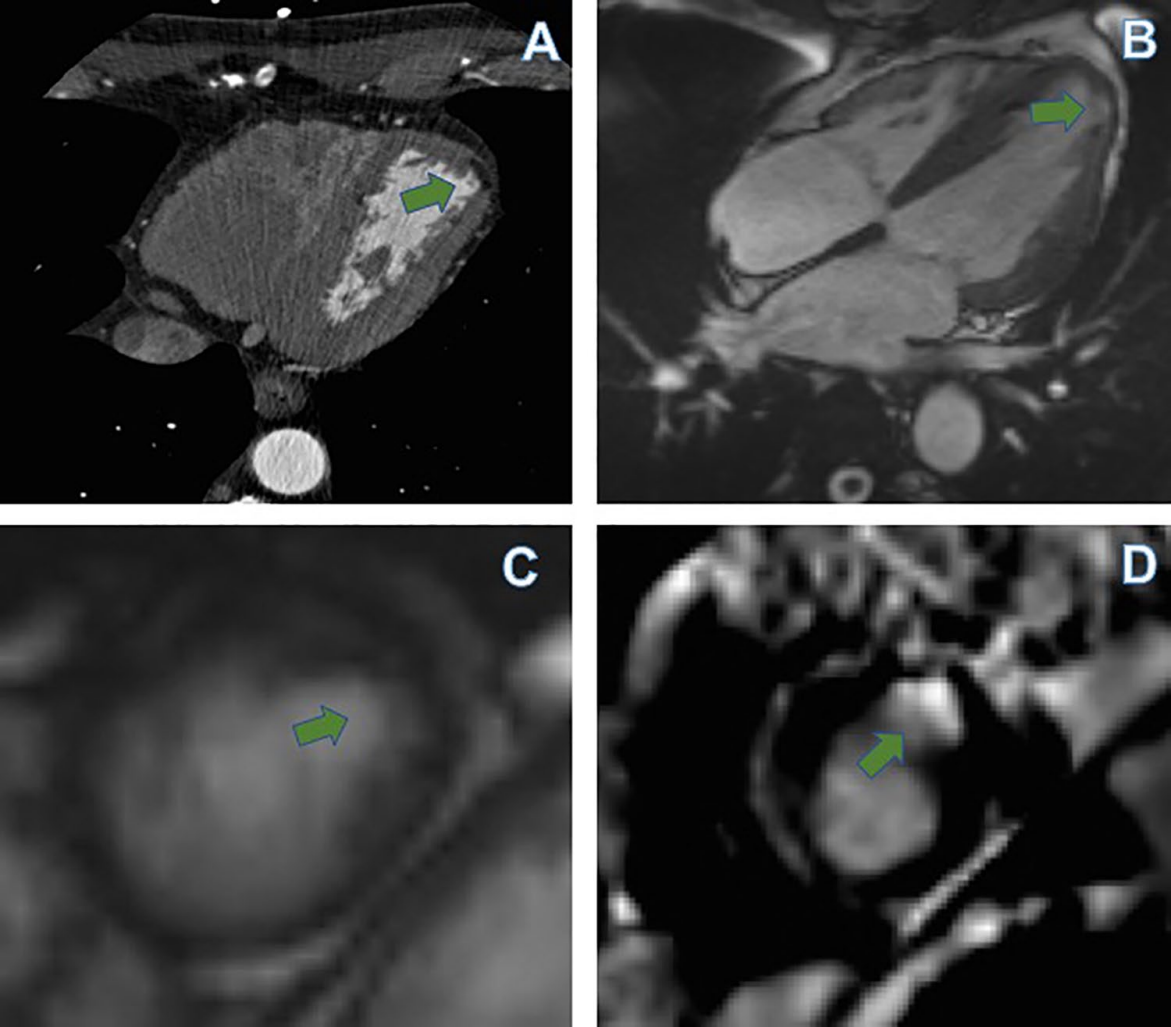
(A) Enhanced cardiac CT axial view shows almost similar but smaller focal aneurysm in the lateral apical segment, (B, C) cardiac MR 4-chamber and short axis views confirm the presence of aneurysm (arrow), and (D) cardiac MR delayed enhancement phase shows abnormal subendocardial enhancement along the same area in the lateral apical segment.

He was admitted and managed as a case of non-ST elevation myocardial infarction. He then underwent an inpatient coronary angiogram, which demonstrated mild coronary artery disease (CAD) and 2 diverticula in the mid anterior wall and larger one in the apical wall on ventriculography (Video). An echocardiogram with contrast revealed an ejection fraction of 64% with no wall motion abnormalities and no diverticulum or aneurysm seen. He had a cardiac Computed Tomography (CT) with contrast which confirmed the presence of mild coronary artery disease with calcified and non-calcified atherosclerotic plaque in the left anterior descending artery (LAD) and possible distal occlusion of a small diagonal branch (Figure 1) with focal aneurysms in the apical lateral and mid septal wall. Cardiac Magnetic Resonance (CMR) was done to further delineate the etiology of these aneurysms and showed 2 discrete areas of ventricular wall thinning corresponding to the regions identified on CT, in addition to delayed enhancement in keeping with scar formation (Figures 2 and 3). The patient was discharged uneventfully on dual anti-platelet therapy for 1 year.

## Discussion

Left ventricular aneurysms are a rare malformation. A thin, scarred well-delineated wall that balloons outward characterizes them, while LV diverticula are outpouchings composed of endocardium, myocardium, and pericardium^1^. Left ventricular aneurysms may occur due to various causes including congenital, inflammatory, and traumatic or may complicate acute coronary syndrome (ACS). The incidence of this complication following ACS is declining due to improved treatment options in the post-interventional era^2^. In addition, the occurrence of multiple left ventricular aneurysms in the setting non-ST elevation myocardial infarction and near normal coronary arteries is uncommon.

In our case, the patient had met the diagnostic criteria for myocardial infarction given his elevated cardiac biomarkers and ischemic chest pain with his angiogram initially demonstrating mild CAD in addition to the presence of an apical diverticulum. As such the diagnosis of MINOCA was made. MINOCA is a syndrome characterized by the presence of myocardial infarction (MI) in the setting of normal or near normal coronary arteries on angiography with luminal stenosis ⩽50%. Causes of MINOCA are broadly divided into epicardial, microvascular dysfunction, and coronary artery embolism^3^. Cardiac CT was instrumental in refuting this diagnosis however, and revealed small diagonal occlusion and a septal branch occlusion from the LAD which probably accounted for the 2 focal apical lateral and mid septal aneurysms respectively.

The CMR proved to be useful in delineating the etiology of his MI demonstrating delayed focal subendocardial enhancement (Figure 3D) in the thinned areas in keeping with scarring and ischemia, as opposed to subepicardial or nodular patchy enhancement which is more suggestive of myocarditis and infiltrative causes such as sarcoidosis. In addition, electrocardiographically the presence of pathological Q waves in the precordial leads may point to the possibility of scar accounting for the mid septal aneurysm. Acquired left ventricular aneurysms due to ischemia usually occur in the setting of more transmural infarction. The occurrence of multiple aneurysms with angiographically near normal coronary arteries is uncommon.

## Conclusion

This case highlights the limitation of coronary angiography in the diagnosis of MINOCA. In addition, it highlights the expanding role of cardiac CT and MR as superior modalities to echocardiography in the diagnosis and further characterization of left ventricular aneurysms. The presence of multiple focal LV aneurysms on LV angiography with mild CAD, should raise the suspicion of small vessel occlusive disease resulting in chronic micro-infarcts, which may account for this rare clinical entity.

## Supplemental Material

sj-pdf-1-cic-10.1177_11795468211006698 – Supplemental material for An Unusual Case of Multiple Left Ventricular Aneurysms Masquerading as Diverticula in the Setting of Myocardial Infarction Click here for additional data file. Supplemental material, sj-pdf-1-cic-10.1177_11795468211006698 for An Unusual Case of Multiple Left Ventricular Aneurysms Masquerading as Diverticula in the Setting of Myocardial Infarction by Hussain Alzayer, Ahmad Alshatti and Akeel Alali in Clinical Medicine Insights: Cardiology
