# Supplementary figures and images for: An Unusual Case of Multiple Left Ventricular Aneurysms Masquerading as Diverticula in the Setting of Myocardial Infarction
Source: Clin Med Insights Cardiol. 2021 Mar 27;15:11795468211006698. doi: 10.1177/11795468211006698 (PMC8842348; doi:10.1177/11795468211006698)

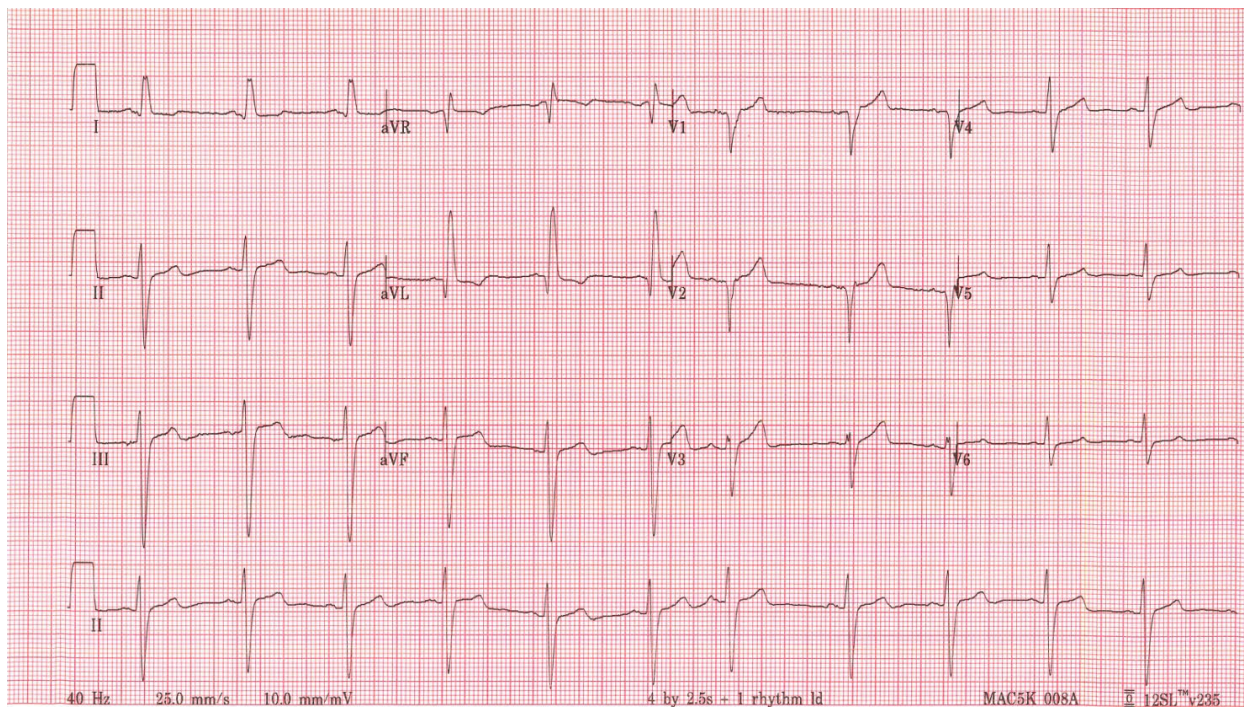

Supplement: sj-pdf-1-cic-10.1177_11795468211006698 – Supplemental material for An Unusual Case of Multiple Left Ventricular Aneurysms Masquerading as Diverticula in the Setting of Myocardial Infarction [file sj-pdf-1-cic-10.1177_11795468211006698.pdf]
